# An Image Fusion Method Based on Sparse Representation and Sum Modified-Laplacian in NSCT Domain

**DOI:** 10.3390/e20070522

**Published:** 2018-07-11

**Authors:** Yuanyuan Li, Yanjing Sun, Xinhua Huang, Guanqiu Qi, Mingyao Zheng, Zhiqin Zhu

**Affiliations:** 1 School of Information and Control Engineering, China University of Mining and Technology, Xuzhou 221116, China; 2 College of Automation, Chongqing University, Chongqing 400044, China; 3 School of Computing, Informatics, and Decision Systems Engineering, Arizona State University, Tempe 85281, AZ, USA; 4 College of Automation, Chongqing University of Posts and Telecommunications, Chongqing 400065, China

**Keywords:** image fusion, sparse representation, NSCT, SML

## Abstract

Multi-modality image fusion provides more comprehensive and sophisticated information in modern medical diagnosis, remote sensing, video surveillance, etc. Traditional multi-scale transform (MST) based image fusion solutions have difficulties in the selection of decomposition level, and the contrast loss in fused image. At the same time, traditional sparse-representation based image fusion methods suffer the weak representation ability of fixed dictionary. In order to overcome these deficiencies of MST- and SR-based methods, this paper proposes an image fusion framework which integrates nonsubsampled contour transformation (NSCT) into sparse representation (SR). In this fusion framework, NSCT is applied to source images decomposition for obtaining corresponding low- and high-pass coefficients. It fuses low- and high-pass coefficients by using SR and Sum Modified-laplacian (SML) respectively. NSCT inversely transforms the fused coefficients to obtain the final fused image. In this framework, a principal component analysis (PCA) is implemented in dictionary training to reduce the dimension of learned dictionary and computation costs. A novel high-pass fusion rule based on SML is applied to suppress pseudo-Gibbs phenomena around singularities of fused image. Compared to three mainstream image fusion solutions, the proposed solution achieves better performance on structural similarity and detail preservation in fused images.

## 1. Introduction

Image fusion generates a composite image via integrating the complementary information from multiple source images in the same scene. The input source images in an image fusion system can be acquired either from various kinds of imaging sensors or from one sensor with different optical parameter settings. So the fused image as output is more fit for human visual perception and machine processing than any single source image. Image fusion techniques have been widely used in computer vision, surveillance, medical imaging, remote sensing, and so on [1].

Pixel-level fusion algorithms are mainly categorized as spatial domain and transform domain based solutions [2]. Spatial domain based solutions directly extract useful information from source images for image fusion [3]. Being the simplest method, pixel weighted average strategy is always applied to source image pixels. It often blurs the contour and edge information of source images, loses the useful information, and causes low-quality image fusion results. To enhance the visual quality of fused image, area and block segmentation based image fusion solutions were proposed [4]. Although the visual performance of fused image is improved, the corresponding segmentation algorithm is comparatively complex, and not good for real-time processing. In spatial-domain based image fusion algorithms, it is difficult to determine the size and features of sub-block. V.Aslantaa proposed a differential evolution algorithm to determine the size of split image [4].

Based on quad-tree structure and morphology, I. De proposed a novel image fusion algorithm [5]. M. Bagher integrated block segmentation and discrete cosine transform into image fusion [6]. Other image block recognition and selection methods, such as pulse-coupled neural networks (PCNNs), artificial neural network [7], had been successfully applied to image fusion. Although most of the existing solutions can obtain high-quality fusion results in certain extents, fused images may still be unsmooth. In transform-domain based fusion methods, source images are transformed into bases and coefficients [2]. The transform coefficients are merged, and then inverted to construct a corresponding fused image. MST methods are widely applied to different image fusion scenes, such as multi-focus [8], infrared-visible [9] and multi-modality medical [2] images fusion. In general, the fusion frameworks based on MST include decomposing source images, merging transform coefficients and reconstructing fused image [2].

Pyramid-based image fusion algorithm is widely used in the transform-domain method. The Laplacian pyramid was used in the multi-scale decomposition of the image, and after that the corresponding scales were merged to form the fused image [2]. Du presented a combination Laplacian pyramid with multiple features for medical image fusion that improved the contrast of the fused image [10]. Jin proposed remote sensing images applied to Baldwinian Clonal Selection Optimization based on pyramid decomposition [11]. This method employed contrast pyramid decomposition in each level of source images, which made the fused image more fit for the human visual system. However, the pyramid-based transform lacks direction, so it cannot extract detailed image information in different directions [7].

Compared to conventional pyramid-based algorithms, wavelet-based transform methods not only contain more temporal and frequency features, and multi-resolution properties, but also achieve good performance in fused results. Mallat first applied wavelet-based transform to image fusion [7]. In recent years, following the continuous research on wavelet analysis and multi-resolution theory, some new wavelet transforms, such as discrete wavelet (DWT) [12], fuzzy wavelet [13], double-tree complex wavelet (DTCWT) [14], and M-band wavelet transform [7], have been introduced into image fusion. It is known that there are some fundamental shortcomings in discrete wavelet transform, like lack of shift invariance and directivity. Because of the shift invariance and directional selectivity of DTCWT on DWT, the artifacts introduced by DWT can be reduced, and the DTCWT is successfully applied to image fusion. However, DWT or DTCWT cannot represent the curve and edge information of the image well [15]. In order to describe the spatial structures of the image more precisely, there are some new multi-scale geometric analysis tools introduced into image fusion. For instance, the inherent geometric structure of the image can be captured by contourlet transform, and maximize the use of geometric characteristics of data, such as line singularities and plane singularities [7]. Since contourlet transform contains the downsampling, it lacks shift invariant property. Nonsubsampled contourlet transform (NSCT) can describe complex spatial structures in various directions well [16]. For conventional MST-based image fusion methods, there are two main weaknesses as follows. One is the loss of contrast. When the weighted average rule is applied to the low-pass fusion, the details of original images are largely lost. The results show that the contrast of fused image is greatly deduced after MST reconstruction. The low-pass band contains most energy of an image. The averaging fusion rule tends to lose partial energy of source images [2]. The other one is that it is difficult to select the decomposition level of MST. When the decomposition level is low, it cannot extract enough spatial details from source images. However, when the decomposition level is high, the high-pass fusion becomes more sensitive to noise and registration. Therefore, it is difficult to make an accurate registration.

As a novel theory of image representation, sparse representation (SR) addresses the natural sparsity of signal, according to the physiological characteristics in the human visual system. SR is a transform-based approach, which is widely used in image classification [17,18], image super-resolution [19], image identification [20], image characteristics extraction [21], image deblurring [22], image target recognition [20,23] and multi-modality information fusion [24]. It was first implemented in image fusion by Li and Yang [25].

An SR-based fusion framework was proposed and a corresponding dictionary for SR was established by discrete cosine transform (DCT). Based on group sparse-representation, Li introduced the de-noising method into medical image fusion [26]. However, as this method has not been corroborated on color medical images, Yang and Liu [27] have proposed several mathematical models for the construction of hybrid dictionaries. The hybrid dictionary can show the specific structures well, but its poor adaptability affects the representation of different types of images. Therefore, a learning-based adaptive dictionary was applied to SR-based image fusion [28]. K-SVD is a classical dictionary training method, which is widely used in SR-based image fusion [29,30]. Based on K-SVD, Yin [24,30] proposed several image fusion methods, such as the multi-focus image fusion method [30] and the multi-modality medical image fusion method [24], which have good state-of-the-art performances and can improve the performance of image details. In order to deal with image fusion on remote sensing, a non-parametric Bayesian [29] adaptive dictionary learning method has been proposed.

The source image can be described by combining the sparse linearity in sparse representation of atoms selected in an over-complete dictionary. The salient information of source images can be represented by only few non-zero elements in sparse coefficients, because the obtained weighted coefficients are sparse. Based on a joint patch clustering, an efficient dictionary learning method was proposed for multi-modality image fusion [31]. Only a few main elements that can effectively describe each joint patch cluster are chosen to construct a compact and informative dictionary. They are combined to form an over-complete dictionary. Multi-modality images are represented by sparse coefficients estimated from the simultaneous orthogonal matching pursuit (SOMP) algorithm. In general, the fusion methods based on SR include three steps. First, each source image is decomposed into many patches by using the sliding window technique (patches are overlapped) directly. Then, it sparsely codes each block to obtain corresponding sparse coefficients. Finally, it merges coefficients into the fused image by the Max-L1 rule.

However, conventional SR-based image fusion methods have the following defects. (1) The fine details of original images such as textures and edges are often smoothed. The reconstructed result is not close to the input signal because all fine details may not be sufficiently represented by dictionary. (2) The consistence of gray in fused image may be caused by the Max-L1 rule, when the original image is captured by different imaging methods. In the past few years, many approaches, such as SR-based and MST-based methods, have been proposed to improve the fusion performance. An image fusion framework that integrates the complementary advantages of MST and SR (MST-SR) was proposed for multi-modality image fusion [31]. It overcomes the shortcomings of MST and SR-based fusion methods at the same time. In K-SVD, the SR-based dictionary learning method is applied to fuse MST low-pass bands. A large number of training images are involved in the iterative learning process of K-SVD. Considering the heavy computational complexity, it has higher costs in practical application, and the dimension of its dictionary is always limited. So this paper presents an image fusion framework called NSCT-SML-SR (NSS) that takes all the complementary advantages of NSCT and SR. First, NSCT decomposes each source image to obtain its high- and low-pass coefficients. With multiple scale and direction characteristics, NSCT can solve the limitations of tradition wavelet methods in the presentation of image curves and edges. Compared to conventional MST-based image fusion method, NSCT has shift-invariant, and suppresses pseudo-Gibbs phenomena. The NSCT-based method is not only convenient to find the relationship among each sub-band of image, but also effectively suppresses mis-registration in fused image. Then it performs Sum Modified-Laplacian (SML) processing over high-pass bands to obtain the pixel metric features of high frequency clear region, which is used in decision-making of image fusion algorithms. The principal component analysis (PCA) dictionary learning algorithm is applied to low-pass bands. Then SR-based fusion algorithm is used to integrate the low-frequency coefficients. The contrast of fused image is improved. The SR-based method is applied to extract the spatial details of low-pass segments. The decomposition in multi-focus image fusion is set to be less than 4 to make the proposed solution more robust to mis-registration. Thus, it is a good solution to solve the problem of decomposition. Therefore, the problem of confirming decomposition level can be well solved. Meanwhile, the expression ability of PCA dictionary satisfies the accuracy of low-frequency component reconstruction, which can prevent the inclination of detail smoothing in the SR-based method. Finally, NSCT inversion of the combined high- and low-frequency coefficients is performed to obtain the fused image.

The key contributions of this paper can be elaborated as the following three points:

1. It decomposes source images into high- and low-pass bands, and applies inverse NSCT to the merged coefficients to obtain fused image. With multi-scale, multi-direction and shift-invariant features, NSCT can suppress pseudo-Gibbs phenomena effectively.
2. It uses PCA-based dictionary learning in the fusion of low-frequency coefficients. It reduces the dimension of learned dictionary and computation time. At the same time, it effectively improves the detailed performance and accuracy of the fused image.
3. It utilizes MAX-SML to fuse high-frequency coefficients. It selects the coefficient with a large SML value as the fusion coefficient. It not only suppresses pseudo-Gibbs phenomena around singularities of the fused image, but also improves the visual quality.

The rest of this paper is organized as follows: Section 2 presents the proposed framework; Section 3 compares the proposed solution with other existing solutions in the fusion of multi-focus, visible-infrared, and medical images; and Section 4 concludes this paper.

## 2. Proposed Framework

**Figure 1:**
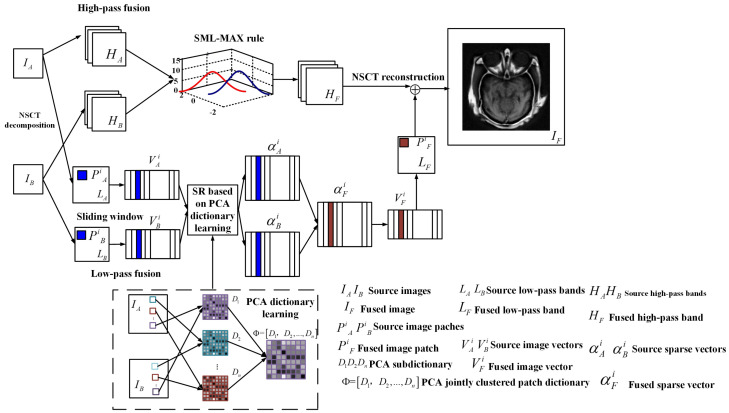
Proposed image fusion framework.

Based on NSCT multi-scale transformation, the proposed NSS fusion framework is shown in Figure 1. To simplify the discussion, it uses two source images for illustration. The proposed framework can be straightforwardly extended to fuse multiple source images.

There are three main steps of the proposed solution; they are as follows:

1. **NSCT Decomposition:** It performs a given NSCT on two original images $\left\{I_{A},I_{B}\right\}$ respectively. The low- and high-pass bands are obtained, which are expressed as $\left\{L_{A},L_{B}\right\}$ and $\left\{H_{A},H_{B}\right\}$ respectively.
2. **Low-pass and High-pass Decomposition:**
  - *Low-pass Decomposition:* It iterates vector generation and sparse representation process for all source image patches ${\left\{P_{A}^{i}\right\}}_{k=1}^{T}$ and ${\left\{P_{B}^{i}\right\}}_{k=1}^{T}$ using Max-L1 rule. The fused result $L_{F}$ of low-pass bands is obtained.
  - *High-pass Decomposition:* It merges high-frequency component $H_{A}$ and $H_{B}$ to obtain the fused high-frequency coefficient $H_{F}$ using the SML-MAX fusion rule.
3. *NSCT Reconstruction:* It does NSCT inversely on $L_{F}$ and $H_{F}$ to reconstruct the final fused image $I_{F}$.

### 2.1. NSCT Transformation

Based on contourlet transform (CT), it proposes nonsubsampled contourlet transform (NSCT) with time shift invariance and direction selectivity. Compared to conventional MST fusion methods, each sub-band image decomposed by NSCT has the same size as the source image, which is easier to use for image fusion.

**Figure 2:**
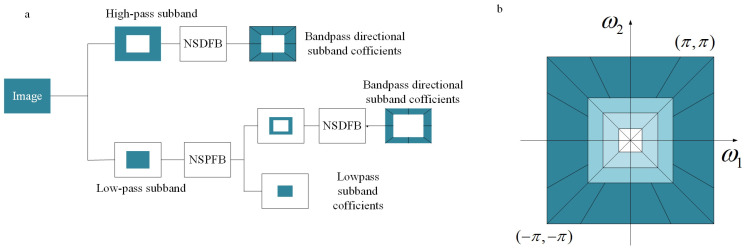
Nonsubsampled contourlet transform (NSCT). (**a**) An overview of proposed NSCT. (**b**) The idealized frequency partitining obtained by NSCT.

CT employs Laplacian Pyramid (LP) and Directional Filter Bank (DFB) in multi-scale and direction decomposition respectively. Based on Nonsubsampled Pyramids Filter Bank (NSPFB) and Nonsubsampled Directional Filter Bank (NSDFB), the proposed NSCT shown in Figure 2 can achieve a rapid expansion with flexible multi-scale, multi-direction and shift invariant. Figure 2a displays an overview of proposed NSCT. Figure 2b illustrates the idealized frequency partitioning obtained by NSCT.

NSPFB is a two-channel Nonsubsampled Filter Bank (NFB), which is used by NSCT. It removes up- and down-samplers from LP, and then does upsampling accordingly. For multi-scale property, it does not need additional filter design.

The decomposition of NSPFB is illustrated by Figure 2a with j=3 stages. The ideal frequency of *j*-stage support region low pass filter is −(ππ2j2j)(ππ2j2j)2. Accordingly, the ideal support of equivalent high-pass filter is the complement of low-pass filter at the corresponding region ${\left[-\left(\pi /2^{j-1}\right),\left(\pi /2^{j-1}\right)\right]}^{2}\backslash {\left[-\left(\pi /2^{j}\right),\left(\pi /2^{j}\right)\right]}^{2}$. The equivalent filters of a *j*-level cascading NSPFB are given by Equation (1).

(1) $$\begin{array}{cc} H_{1}\left(z^{2^{n-1}I}\right) & \Pi_{j=0}^{n-2}H_{0}\left(z^{2^{j}I}\right),1\leq n\leq J\\ & \Pi_{j-0}^{n-2}H_{0}\left(z^{2^{j}I}\right),n=J+1 \end{array}$$

where $H_{0}\left(z\right)$ and $H_{1}\left(z\right)$ denote the low-pass filter and the corresponding high-pass filter respectively.

NSDFB used by NSCT is a shift-invariant version of the dual-channel critical sampling DFB in CT. Therefore, the two-dimensional frequency plane is divided into a directional wedge by the results in tree structured filter banks.

The upper sampling fan filters $U_{j}\left(z^{D}\right)$ (j=0,1) have chessboard frequency support, where the sampling matrix *D* is a quincunx matrix i.e.,

$$D=\left[\begin{array}{cc} 1 & -1\\ 1 & 1 \end{array}\right],$$

The equivalent filter in each channel $U_{k}\left(z\right)\left(k=0,1,2,3\right)$ can be obtained by Equation (2). Although the higher sampling matrix is more complex, the higher level directional decomposition also follows a similar strategy. Thus, a single NFB fan filter can supply all filter banks in the NSDFB tree structure. Moreover, the usage of upsampled filters for filtering does not increase the computational complexity. Filters in the NSDFB tree have the same complexity as that in fan NFB.

(2) $$U_{k}\left(z\right)=U_{i}\left(z\right)U_{j}\left(z^{D}\right)$$

As shown in Figure 2a, NSCT can be obtained by integrating NSPFB and NSDFB. The 2-D two channel nonsubsampled filter banks are the core of NSCT. The perfect reconstruction of the two-channel unsampled filter banks can be realized, in which the filter satisfies the Bezout identity, as shown in Equation (3).

(3) $$H_{0}\left(z\right)G_{0}\left(z\right)+H_{1}\left(z\right)G_{1}\left(z\right)=1$$

where $H_{0}\left(z\right)$ and $H_{1}\left(z\right)$ both represent the decomposition filters. $G_{0}\left(z\right)$ and $G_{1}\left(z\right)$ show the synthesis filters. If the two-channel nonsubsampled filter banks in both NSDFB and NSPFB satisfy the Bezout identity and are invertible, NSCT is invertible. NSCT is flexible. It allows for an arbitrary number of directions at each scale; in particular, it satisfies the anisotropic scaling law [32,33] as a key quality for the expansion of nonlinear approximate behavior [9,34].

### 2.2. Low-Pass Fusion

#### 2.2.1. Dictionary Learning

The main difficulty of image reconstruction and fusion techniques is choosing an over-complete dictionary based on sparse representation. Over-complete dictionaries use DCT and wavelet in most cases. A fixed dictionary is easy to be achieved; however, its performance is somewhat limited in terms of data and application types. Aharon [31] developed a dictionary-learning based method to make the dictionary adapt to different input image data [31]. A dictionary called K-SVD is learned from a set of training images and updated adaptively by SVD operation. Comparing with other fixed dictionary based methods, K-SVD has better performance in many image reconstruction approaches. Elad and Aharon proved that adaptive dictionaries from noisy input images are sometimes superior to fixed DCT or global training dictionaries [31]. K-SVD is an iterative learning process, which is widely used in a large number of training images in practical applications with high cost. Due to the high computational complexity, the dimension of learned dictionary is constrained [27]. The lessons obtained by different dictionary learning methods and the clustering method are used as motivation. To solve the problem of multi-modal image fusion, a resultful dictionary learning method based on joint block clustering is selected. More diverse features can be supplied by images from multiple sensors. Therefore, a complete dictionary can be formed by clustering similar patches from all source images. The trained dictionaries can be combined together to describe all multi-modal image signals by adding patches from all source images. In addition, the over-complete dictionary of K-SVD has a high structure. All multi-modal image signals can be described by a learning dictionary, but it is redundant. By eliminating redundancy in learned dictionaries (reducing the dictionary size) [35], the computational complexity of proposed image fusion can be reduced.

**Figure 3:**
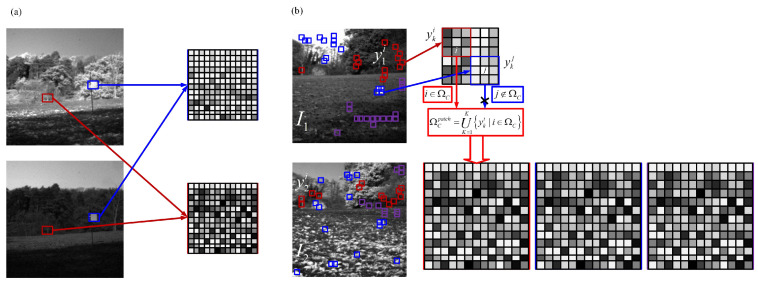
(**a**) Illustration of generating local patch dictionaries. (**b**) Illustration of generating joint patch clusters.

An over-complete dictionary constructed by clustering all local neighbor patches is shown in Figure 3a. Figure 3b demonstrates a dictionary constructed by those patches from joint patch clusters directly. More image patches from source images are involved, so it may cause redundancy in the learned dictionary. Recently, some methods have been proposed to reduce the size of over-complete dictionaries or to utilize compact dictionaries [36,37]. The proposed solution combines the main components of each joint patch cluster to learn a compact and informative dictionary. Since several PCA bases can be well approximated to several cluster patches, select the most useful *p* principal components to form a sub-dictionary as follows:

(4) $$D_{C}=\left[d_{1},\ldots d_{p}\right]s.t.p=\arg\max_{p}\left\{\sum_{j=p+1}^{n}L_{j}>\delta \right\}$$

where $D_{C}$ expresses the sub-dictionary of the Cth cluster, which is composed of *p* eigenvectors, i.e., atom. The eigenvalues of *j*th eigenvector $d_{j}$ correspond to $L_{j}$. Ranking eigenvalues in descending order (i.e., $L_{1}>L_{2}>\ldots >L_{n}$). δ is a parameter to control the amount of approximation with rank *p*. If δ is set too high, the constructed sub-dictionary may have an insufficient number of atoms. In this way, the signals reconstructed by using such dictionaries become too smooth. Thus, it should at least be wise to determine δ to get the minimum number of atoms that correctly represent the signal. In this paper, we set δ=0.95. The typical PCA bases are chosen as the atoms of the corresponding sub-dictionaries, which can best describe the underlying structure of each cluster. Once all sub-dictionaries of all joint patch clusters are obtained, they are combined to form a single dictionary as follows:

(5) $$\Phi =\left[D_{1},D_{2},\ldots ,D_{C}\right]$$

This combination dictionary Φ is called the aggregate patches dictionary in (5) because it contains cluster principal components across input source images. Compared with the fixed DCT dictionary and the k-svd learning dictionary, the dictionary based on principal component analysis (PCA) presented in this paper is more compact, but the most informative components are still included in the joint patch clusters. Therefore, it attains the good reconstruction performance by reducing the substantial computation costs.

#### 2.2.2. Low-Pass Fusion

A sparse-representation based fusion approach is applied to each low-pass NSCT band. The fusion scheme contains the following steps:

1. $L_{A}$ and $L_{B}$ are divided into image patches of size $\sqrt{n}\times \sqrt{n}$ by using sliding window technique with steps of s pixels from the top left to the lower right corner. Suppose there are **T** patches denoted as ${\left\{P_{A}^{i}\right\}}_{k}^{T}$ and ${\left\{P_{B}^{i}\right\}}_{k}^{T}$ in $L_{A}$ and $L_{B}$ respectively.
2. For each position **i**, rearrange $\left\{P_{A}^{i},P_{B}^{i}\right\}$ into column vectors $\left\{v_{A}^{i},v_{B}^{i}\right\}$. Then normalize each vector’s mean value to zero to obtain $\left\{V_{A}^{i},V_{B}^{i}\right\}$ by
  (6) $$V_{A}^{i}=v_{A}^{i}-\overset{-}{v_{A}^{i}}\cdot \left[\begin{array}{c} 1\\ 1\\ \begin{array}{c} .\\ .\\ . \end{array}\\ 1 \end{array}\right]$$
  where $\left[\begin{array}{c} 1\\ 1\\ \begin{array}{c} .\\ .\\ . \end{array}\\ 1 \end{array}\right]$ denotes an n×1 vector. $\overset{-}{v_{A}^{i}}$ is the mean values of all the elements in $v_{A}^{i}$. $V_{B}^{i}$ is obtained in the same way as $V_{A}^{i}$.
3. Calculate the sparse coefficient vectors $\left\{\alpha_{A}^{i},\alpha_{B}^{i}\right\}$ of $\left\{V_{A}^{i},V_{B}^{i}\right\}$ using Simultaneous Orthogonal Matching Pursuit (SOMP) algorithm by
  (7) $$\alpha_{A}^{i}=\underset{\alpha_{A}^{i}}{\arg\min}{∥\alpha ∥}_{0}\;s.t.{∥y_{A,L}^{i}-\Phi \alpha ∥}_{2}^{2}<\varepsilon$$
  where ε represents the bounded representation error, ${∥\alpha ∥}_{0}$ represents a count of non-zero items in α, $y_{A,L}^{i}$ denotes the low frequency component $L_{A}$. Φ is the learned dictionary. Similarly, $\alpha_{B}^{i}$ can also be obtained using Equation (7).
4. Merge $\alpha_{A}^{i}$ and $\alpha_{B}^{i}$ by Max-L1 rule to obtain fused sparse coefficients.
  (8) $$\alpha_{F}^{i}=\left\{\begin{array}{c} \alpha_{A}^{i},if{∥\alpha_{A}^{i}∥}_{1}>{∥\alpha_{B}^{i}∥}_{1}\\ \alpha_{B}^{i},otherwise \end{array}\right.$$
  The fused result of $v_{A}^{i}$ and $v_{B}^{i}$ is calculated by
  (9) $$V_{F}^{i}=\Phi \alpha_{F}^{i}+\overset{-}{v_{F}^{i}}\cdot 1$$
  where the merged mean value $\overset{-}{v_{F}^{i}}$ is obtained by
  (10) $$\overset{-}{v_{F}^{i}}=\left\{\begin{array}{c} \overset{-}{v_{A}^{i},}if\alpha_{F}^{i}=\alpha_{A}^{i}\\ \overset{-}{v_{B}^{i}},otherwise \end{array}\right.$$
5. All the original image patches in ${\left\{P_{A}^{i}\right\}}_{k}^{T}$ and ${\left\{P_{B}^{i}\right\}}_{k}^{T}$ are iterated according to the above (2) to (4) processes to obtain all the fused vectors ${\left\{V_{A}^{i}\right\}}_{i=1}^{T}$.For each $V_{F}^{i}$, it is reshaped into a $\sqrt{n}\times \sqrt{n}$ size patch $P_{F}^{i}$. Then plug $P_{F}^{i}$ into its original position in $L_{F}$. The low-pass fused result $L_{F}$ is obtained by averaging the accumulation times of each pixel’s value in $L_{F}$.

### 2.3. High-Pass Fusion

In this paper, the high frequency coefficients are fused using the MAX-SML rule. Select the coefficient with a large SML value as the fusion coefficient. Suppress pseudo-Gibbs phenomena around singularities of fused image. The fusion scheme contains the following steps:

1. NSDFB decomposes each high-frequency component $H_{A}$ and $H_{B}$ obtained by NSCT decomposition to obtain the coefficients $I_{A}^{l,k}\left(i,j\right)$ and $I_{B}^{l,k}\left(i,j\right)$ at (*i*, *j*) in the *1*-scale *k*-direction.
2. Calculate the Modified Laplacian (ML) and SML of the high-frequency coefficients $I_{A}^{l,k}\left(i,j\right)$ and $I_{B}^{l,k}\left(i,j\right)$. The ML and SML are defined as follows:
  (11) $$\begin{array}{c} ML^{l,k}\left(i,j\right)=\left|2I^{l,k}\left(i,j\right)-I^{l,k}\left(i-step,j\right)-I^{l,k}\left(i+step,j\right)\right|+\\ \left|2I^{l,k}\left(i,j\right)-I^{l,k}\left(i,j-step\right)-I^{l,k}\left(i,j+step\right)\right| \end{array}$$
  where the step denotes the variable spacing between different coefficients. In this paper, we set step = 1.
  (12) $$SML^{l,k}\left(i,j\right)=\sum_{p=-P}^{P}{\sum_{q=-Q}^{Q}\left[ML^{l,k}\left(i+p,j+q\right)\right]}^{2}$$
  where *P* and *Q* denote the window of size (2P+l)×(2Q+l).
3. Merge $I_{A}^{l,k}\left(i,j\right)$ and $I_{B}^{l,k}\left(i,j\right)$ by the "SML-MAX" rule to obtain the high-frequency fused coefficient.
  (13) $$I_{F}^{l,k}\left(i,j\right)=\left\{\begin{array}{c} \begin{array}{cc} I_{A}^{l,k}\left(i,j\right), & if:SML_{A}^{l,k}\left(i,j\right)\geq SML_{B}^{l,k}\left(i,j\right) \end{array}\\ \begin{array}{cc} I_{B}^{j,k}\left(i,j\right), & if:SML_{A}^{l,k}\left(i,j\right)<SML_{B}^{j,k}\left(i,j\right) \end{array} \end{array}\right.$$
  where $SML_{A}^{l,k}\left(i,j\right)$ and $SML_{B}^{l,k}\left(i,j\right)$ are the SML clarity of the high-frequency coefficients $I_{A}^{l,k}\left(i,j\right)$ and $I_{B}^{l,k}\left(i,j\right)$ respectively. $I_{F}^{l,k}\left(i,j\right)$ denotes the fused high-frequency coefficient at (*i*, *j*) in the *1*-scale *k*-direction.
4. Iterate the above (2) and (3) process for all the high-frequency coefficients in high-frequency component $H_{A}$ and $H_{B}$ to obtain the high-frequency fused coefficient $H_{F}$.

The final fused image $I_{F}$ is reconstructed by performing the corresponding inverse NSCT over $L_{F}$ and $H_{F}$.

## 3. Experiments and Analysis

### 3.1. Experiment Preparation

**Figure 4:**
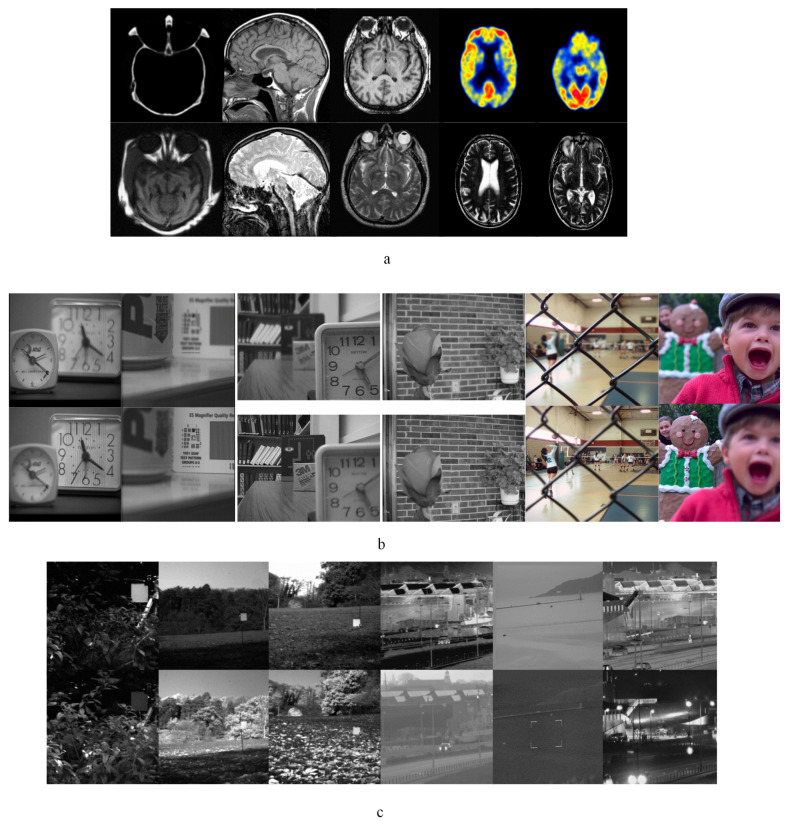
Source images of comparative experiments: (**a**) Medical image pairs. (**b**) Multi-focus image pairs. (**c**) Infrared-visible image pairs.

In our experiments, 34 sets of medical images, 29 sets of multi-focus and 8 sets of infrared-visible images are applied to the fusion performance testing respectively. The resolution of test images are 256 × 256, 240 × 320 and 520 × 520 respectively. Parts of representative images are shown in Figure 4. In Figure 4, image pairs (a), (b), and (c) show image sets of medical, multi-focus, and infrared-visible respectively. Medical image sets are acquired from *http://www.med.harvard.edu/aanlib/home.html*. Infrared-visible and gray-level multi-focus image pairs were collected by Liu [2] and can be downloaded from *quxiaobo.org*. The color multi-focus image sets are from Lytro-multi-focus data-set at *http://mansournejati.ece.iut.ac.ir*. All the experiment’s program’s codes are programmed in Matlab 2014a on an Intel(R) Core(TM)i7-4790CPU @ 3.60GHz Desktop with 8.00 GB RAM.

#### Objective Evaluation Metrics

In evaluate the quality of fused images, single evaluation metric lacks adequacy and objectivity. Therefore, a plurality of evaluation metrics are used to comprehensively evaluate the image quality as necessary. In this paper, eight evaluation metrics are applied to objectively evaluate the fusion performance of different fusion approaches, which are $Q^{TE}$ [38,39], $Q^{IE}$ [38,40], $Q^{AB/F}$ [41], $Q^{P}$ [38,42], MI [43], $Q^{Y}$ [38,40], $Q^{CB}$ [38,44], and VIF [45].

$Q^{TE}$ [38,39] and $Q^{IE}$ [38,40] evaluate the Tsallis entropy and nonlinear correlation information entropy of fused images respectively. $Q^{AB/F}$ [41] and $Q^{P}$ [38,42] are used to measure the edge information. $Q^{AB/F}$ [41] is a gradient-based quality index, and $Q^{P}$ [38,42] is an image fusion performance metric based on phase consistency. MI [38] and $Q^{Y}$ [38,40] are metrics for evaluating the similarity between the fused image and source images.

MI [38] measures the degree of interdependence between two variables, and $Q^{Y}$ [38,40] evaluates the structural similarity between two variables. $Q^{CB}$ [38,44] and VIF [45] evaluate the human visualisation performance of fused images. $Q^{CB}$ [38,44] is human perception inspired fusion metric. VIF [45] is defined as the ratio between fused image information and source images information.

### 3.2. Experiment Results of Four Popular Fusion Methods

In this subsection, four popular fusion methods are applied to demonstrate the advantages of proposed image fusion framework (NSS). The rest of the popular fusion methods include Kim’s multi-modal image fusion proposed by Minjae Kim [46], the novel multi-modal image fusion method based on image cartoon-texture (CT) decomposition proposed by Zhu [47], and the MST- and SR-based image fusion framework proposed by Liu [2].

In this experiment, we set the NSCT filters mainly based on the optimal setting obtained by Liu [2]. We use the “9-7” as the NSPFB and the "pkva" as the NSDFB. Furthermore, the directional number of the four decomposition levels is set to 4, 8, 8 and 16 in order. For the NSS fusion method, the image patch decomposition size is set to 8 × 8. The dictionary use in sparse model is learned by PCA method.

The dictionary used in sparse model is learned by PCA method.

#### 3.2.1. Experiment Results of Medical Images

In modern medical diagnosis, various types of medical images provide great help in the accurate diagnosis of diseases. Common medical image techniques include X-Ray, Computed Tomograpy (CT), Magnetic Resonance (MR), and Positron Emission Tomograph (PET), etc. There are significant differences in the attention of different modal medical images of human organs and tissues, because of the different imaging mechanisms. Single-type image often fails to provide comprehensive and sufficient information for disease diagnosis. Clinically, doctors generally need to synthesize multiple different types of medical images from the same position to diagnose patient’s condition, which often brings great inconvenience and affects the accuracy of diagnosis. As a solution to these issues, the multi-modal medical image fusion is successfully applied to medical diagnosis. As a key advantage, multi-modal medical image fusion combines the information from different modalities of medical images and presents the combined one in a fused image.

**Figure 5:**
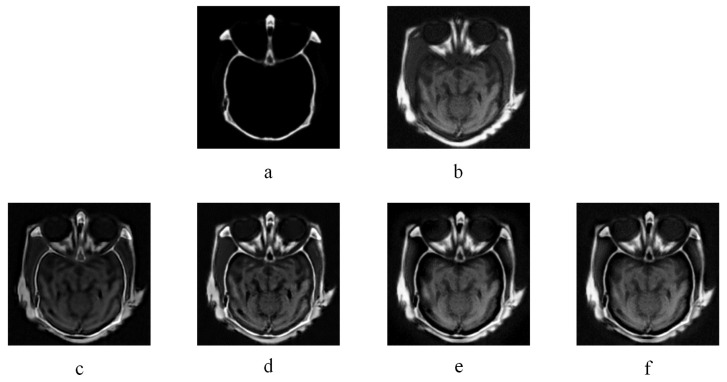
CT image fusion experiments, (**a**) and (**b**) are two CT source images. (**c**) KIM. (**d**) Zhu. (**e**) Liu. (**f**) NSS.

**Figure 6:**
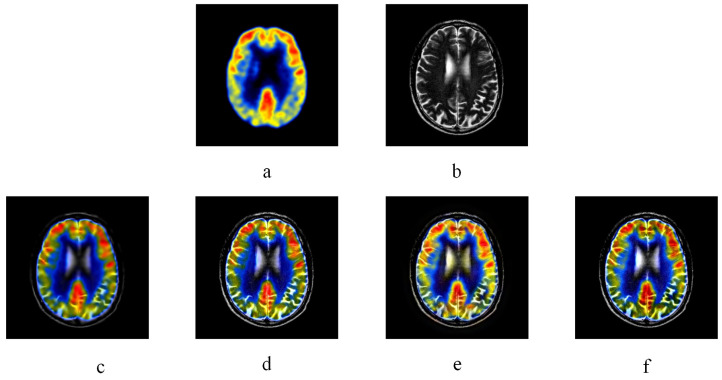
CT-MR image fusion experiments, (**a**) and (**b**) are CT and MR source image respectively. (**a**) KIM. (**b**) Zhu. (**e**) Liu. (**f**) NSS.

Figure 5 and Figure 6 are two multi-modal medical image fusion examples. In Figure 5 and Figure 6, (a) and (b) are two source images, and (c), (d), (e), and (f) illustrate the fused results of KIM, CT, MST-SR, and NSS image fusion method respectively. The different organ information of the human body is reflected by captured images from different modalities. As illustrated in Figure 5 and Figure 6, comparing with the fused images of KIM and CT methods, the fused images obtained by the NSS method have clearer details of the edges and better contrast. Compared to the MST-SR method, the NSS method performs excellently in detail preservation. For example, in the middle white area of Figure 5 obtained by the MST-SR method, some minor details are lost, and the color of Figure 6 obtained by the MST-SR method is unnatural.

Table 1 lists the objective assessments of four popular fusion methods on medical images. As shown in Table 1, compared to other methods, the fusion image obtained by the NSS method outperforms in several evaluation metrics, $Q^{IE}$, $Q^{AB/F}$, MI, $Q^{Y}$ and $Q^{CB}$. From the properties of these evaluation indicators, it can be indicated that fused image obtained by the NSS approach has excellent similarity with source images, and better preserves details. Although, $Q^{TE}$ and $Q^{P}$ metrics are not as good as the CT method, and the VIF metric is a bit lower than the MST-SR method, the NSS fusion framework performs excellently on the preservation of details. In summary, NSS achieves better fusion effects in multi-modal medical image fusion.

#### 3.2.2. Experiment Results of Multi-Focus Images

Since the optical system has a limited range of focus, getting a full-focus image in one scene is difficult to achieve. When an object is within the focus range, a clear image can be captured. But the out-of-focus objects suffer from blurs in various degrees. At present, the resolution of the optical lens is continuously improving. However, the overall impact of limited focusing range on imaging still exists. However, a fully focused image in one scene can be obtained by multi-focus image fusion. The multi-focus fusion techniques can effectively overcome the limited focus range of the optical system [1,27]. Multi-focus fusion techniques can effectively overcome the limited focus range of the optical system to achieve a fully focused image obtainment in one scene. Furthermore, it lays the foundation for image processing, such as feature extraction.

**Figure 7:**
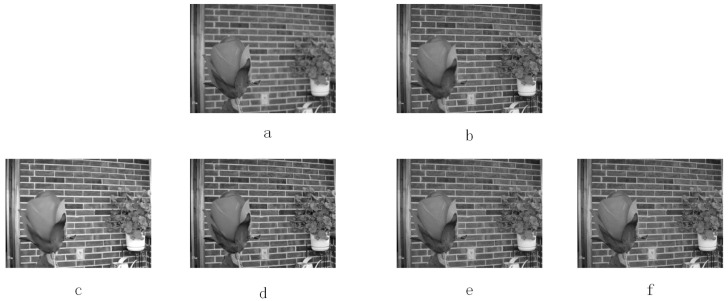
Multi-focus image fusion experiments. (**a**) and (**b**) are two multi-focus source images. (**c**) KIM. (**d**) Zhu. (**e**) Liu. (**f**) NSS.

**Figure 8:**
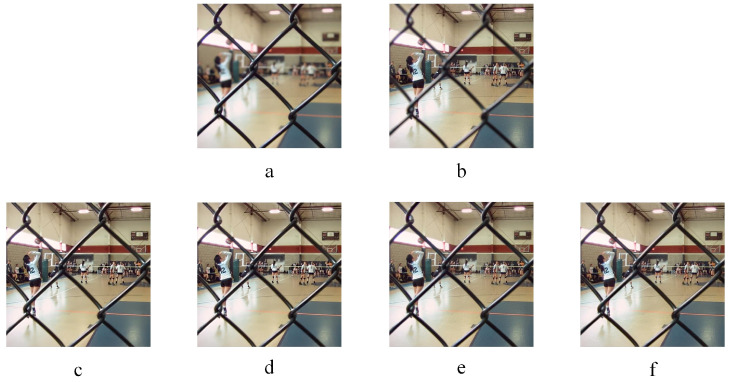
Multi-focus color image fusion experiments. (**a**) and (**b**) are two multi-focus color source images. (**c**) KIM. (**d**) Zhu. (**e**) Liu. (**f**) NSS.

Figure 7 and Figure 8 show the multi-focus image fusion of black-white and color images respectively. Figure 7 and Figure 8a–b are source images. Figure 7 and Figure 8c–f represent the fused images obtained by KIM, CT, MST-SR, and NSS method respectively. As shown in Figure 7, petal texture details of images fused by KIM and CT approaches are poorer than those of the MST-SR and NSS methods. For the two fused images obtained by the MST-SR and NSS methods, the quality of fusion shown in the human visual system is equally good. It is difficult to distinguish these two fusion results. Similarly, in Figure 8, since the quality of four fused images is equally good in the human visual system, it is difficult for the human visual system to distinguish between the four fused images. Therefore, the objective measurement is a method for better measuring the fusion performance.

As illustrated in Table 2, the six evaluation metrics of the NSS approach, $Q^{TE}$, $Q^{IE}$, $Q^{P}$, MI, $Q^{Y}$, $Q^{CB}$, all have excellent performance. It can be inferred that the NSS fusion framework performed excellently in the preservation of information and details. Moreover, for the human visual performance evaluation metric $Q^{CB}$, the proposed method shows good performance. MST-SR obtains the highest score on $Q^{AB/F}$ and VIF, it means that the edge retention and human visualisation performance are better than the NSS method. Although $Q^{AB/F}$ and VIF of the NSS method are slightly small, this fusion approach performs excellently in the preservation of information and details, and it performs good in visualisation and edge retention.

#### 3.2.3. Experimental Results of Infrared-Visible Images

In the reconnaissance shooting task, visible light cameras and infrared imaging devices are used to acquire the object images. Infrared thermography uses thermal radiation technique to convert infrared wavelengths beyond the human eye observation wavelength into visible information mapped into the image. However, the obtained images have poor contrast and cannot extract enough details. Images obtained by visible light camera have high resolution and enough detailed information of texture and edge. However, the imaging quality is easily affected by natural conditions. Since the differences and limitations between visible light and infrared images [48], relying solely on a single type of image is difficult to meet the actual needs of the project. The infrared-visible image fusion technique offers the full use of complementary information and spatial-temporal correlation of visible and infrared images to better meet engineering requirements. Images with high quality and comprehensive information are obtained by fusing multiple image information [49].

**Figure 9:**
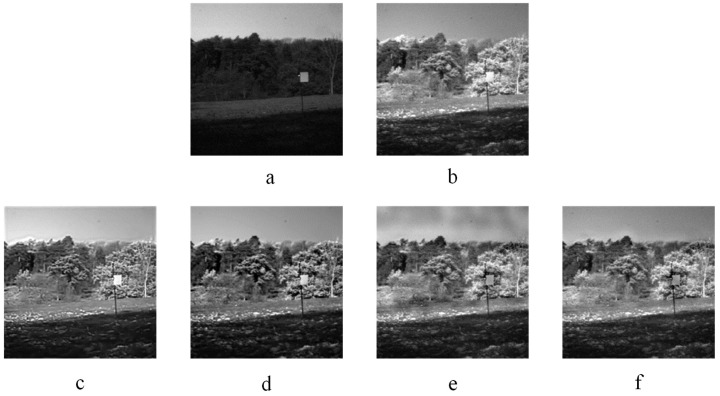
Infrared-visible image fusion experiments-1. (**a**) and (**b**) are two infrared-visible source images. (**c**) KIM. (**d**) Zhu. (**e**) Liu. (**f**) NSS.

**Figure 10:**
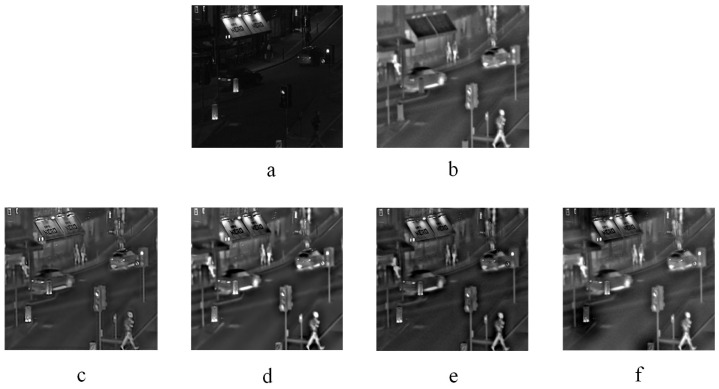
Infrared-visible image fusion experiments-2. (**a**) and (**b**) are two infrared-visible source images. (**c**) KIM. (**d**) Zhu. (**e**) Liu. (**f**) NSS.

Two infrared-visible image fusion examples are displayed in Figure 9 and Figure 10. Figure 9 and Figure 10a–b are source images. Figure 9 and Figure 10c–f represent the fused images obtained by the KIM, CT, MST-SR and NSS methods respectively. As indicated in Figure 9, the image fused by the KIM approach has too high brightness of plants edge and poor texture details. In the image fused by the MST-SR algorithm, the distribution of the sky grayscale is fair. The fused images obtained by the CT and NSS methods get good performance on details and grayscale. In Figure 10, the fused image of KIM does not show as good of contrast as NSS and CT methods. The sharpness of MST-SR and KIM fused images is poorer than that of the NSS approach. Additionally, the pedestrian’s detailed information also shows that the CT and NSS methods perform excellently in image details contrast and brightness.

As illustrated in Table 3, the image fused by the CT method obtained the best $Q^{P}$ score. However, the human visualisation performance and details preservation of image fused by the CT method are poorer than that of the NSS approach. The NSS method obtains the highest $Q^{TE}$, $Q^{TE}$, $Q^{AB/F}$, MI, $Q^{CB}$ and VIF. It can be indicated that the NSS approach performs excellently in visual quality and in the preservation of structural, and performs good in edge retention. Therefore, the NSS fusion framework is superior to other methods.

Based on the above experiments, the effectiveness of the proposed NSS image fusion framework is verified via the subjective visual effects and objective evaluation indicators.

## 4. Conclusions

This paper talks about multi-source image fusion, which covers multi-modal medical, multi-focus, and visible-infrared image fusion. An image fusion framework (NSS), based on NSCT and SR, is proposed to solve the contrast loss, the difficulties in the selection of decomposing level, the defects of representation ability, and other weaknesses in conventional MST and SR-based fusion algorithms. In this fusion framework, low-frequency coefficients are fused via the SR-based scheme which uses PCA dictionary learning, and the high-frequency coefficients are fused via the Sum Modified-laplacian (SML) rule. The fused image is obtained by performing inverse transform of NSCT on fusion coefficients. Compared with conventional MST fusion methods, the NSS framework can obtain more informative fused images. The NSS framework can also improve the detailed performance and accuracy of the fused image by using PCA dictionary learning in comparison with conventional SR-based methods. It eliminates the gray-scale discontinuity, while effectively preventing smooth fine details by sliding window technology. Experiment results prove that the NSS fusion approach sufficiently integrates the advantages of NSCT and SR. Compared with three mainstream fusion methods, the NSS method can achieve superior performance in fused results. Particularly, the NSS method owns the advantages of simple implementation, high efficiency and good performance. It owns the great application prospect in infrared-visible image fusion. Furthermore, the proposed fusion framework also has great potential in the visible-infrared image fusion. In the future, we will improve the computational efficiency and enhance the image fusion performance. Particularly, the NSS fusion framework will be continuously optimized for visible-infrared image fusion.

## Figures and Tables

**Table 1:** Objective evaluations of medical image fusion experimentation.

	$Q^{\mathrm{TE}}$	$Q^{\mathrm{IE}}$	$Q^{\mathrm{AB}/F}$	$Q^{P}$	MI	$Q^{Y}$	$Q^{\mathrm{CB}}$	VIF
KIM	0.5241	0.8063	0.3564	0.2710	1.8923	0.4701	0.5078	0.2584
CT	**0.5544**	0.8069	0.5230	**0.4895**	2.0000	0.6180	0.5465	0.2958
MST-SR	0.5125	0.8067	0.5812	0.3975	1.9624	0.6574	0.5324	**0.3174**
NSS	0.5371	**0.8070**	**0.6148**	0.4430	**2.0093**	**0.6874**	**0.5482**	0.3081

The bolded number is the maximum value of each column.

**Table 2:** Objective evaluations of multi-focus image fusion experimentation.

	$Q^{\mathrm{TE}}$	$Q^{\mathrm{IE}}$	$Q^{\mathrm{AB}/F}$	$Q^{P}$	MI	$Q^{Y}$	$Q^{\mathrm{CB}}$	VIF
KIM	0.7486	0.8208	0.6521	0.7572	4.1530	0.8126	0.6969	0.7887
CT	0.7494	0.8208	0.6943	0.7928	3.9241	0.8442	0.6829	0.7677
MST-AR	0.8912	0.8276	**0.8370**	0.8380	4.6436	0.9561	0.7718	**0.8204**
NSS	**0.9174**	**0.8294**	0.8260	**0.8441**	**4.7723**	**0.9596**	**0.7767**	0.8151

The bolded number is the maximum value of each column.

**Table 3:** Objective evaluations of infrared-visible image fusion experimentation.

	$Q^{\mathrm{TE}}$	$Q^{\mathrm{IE}}$	$Q^{\mathrm{AB}/F}$	$Q^{P}$	MI	$Q^{Y}$	$Q^{\mathrm{CB}}$	VIF
KIM	0.5121	0.8119	0.6849	0.6046	2.6497	0.8014	0.5883	0.4678
CT	0.5014	0.8102	0.7167	**0.7263**	2.5742	0.8338	0.5885	0.4919
MST-SR	0.6215	0.8175	0.8164	0.6966	3.2251	0.9189	0.6842	0.5267
NSS	**0.6573**	**0.8177**	**0.8342**	0.7207	**3.3989**	**0.9332**	**0.6861**	**0.5376**

The bolded number is the maximum value of each column.

## References

[1] Li H., Li X., Yu Z., Mao C. (2016). Multifocus image fusion by combining with mixed-order structure tensors and multiscale neighborhood. Inf. Sci..

[2] Liu Y., Liu S., Wang Z. (2015). A general framework for image fusion based on multi-scale transform and sparse representation. Inf. Fusion.

[3] Li H., He X., Tao D., Tang Y., Wang R. (2018). Joint medical image fusion, denoising and enhancement via discriminative low-rank sparse dictionaries learning. Pattern Recognit..

[4] Aslantas V., Kurban R. (2010). Fusion of multi-focus images using differential evolution algorithm. Expert Syst. Appl..

[5] De I., Chanda B. (2013). Multi-focus image fusion using a morphology-based focus measure in a quad-tree structure. Inf. Fusion.

[6] Haghighat M.B.A., Aghagolzadeh A., Seyedarabi H. (2011). A non-reference image fusion metric based on mutual information of image features. Comput. Electr. Eng..

[7] Li S., Kang X., Fang L., Hu J., Yin H. (2017). Pixel-level image fusion: A survey of the state of the art. Inf. Fusion.

[8] Li H., Liu X., Yu Z., Zhang Y. (2016). Performance improvement scheme of multifocus image fusion derived by difference images. Signal Process..

[9] Li H., Qiu H., Yu Z., Zhang Y. (2016). Infrared and visible image fusion scheme based on NSCT and low-level visual features. Infrared Phys. Technol..

[10] Du J., Li W., Xiao B., Nawaz Q. (2016). Union Laplacian pyramid with multiple features for medical image fusion. Neurocomputing.

[11] Jin H., Xing B., Wang L., Wang Y. (2015). Fusion of remote sensing images based on pyramid decomposition with Baldwinian Clonal Selection Optimization. Infrared Phys. Technol..

[12] Smith L.C., Turcotte D.L., Isacks B.L. (1998). Stream flow characterization and feature detection using a discrete wavelet transform. Hydrol Process..

[13] Yilmaz S., Oysal Y. (2010). Fuzzy wavelet neural network models for prediction and identification of dynamical systems. IEEE Trans. Neural Netw..

[14] Mavudila R., Masmoudi L., Cherkaoui M., Hassanain N. (2012). MRI images de-noising based in Dual-Tree complex Wavelet and Bayesian MAP Estimator. Int. J. Mod. Eng. Res..

[15] Li H., Yu Z., Mao C. (2016). Fractional differential and variational method for image fusion and super-resolution. Neurocomputing.

[16] Upla K.P., Joshi M.V., Gajjar P.P. (2015). An edge preserving multiresolution fusion: Use of contourlet transform and MRF prior. IEEE Trans. Geosci. Remote Sens..

[17] Cao J., Hao J., Lai X., Vong C.M., Luo M. (2016). Ensemble extreme learning machine and sparse representation classification. J. Franklin Inst..

[18] Liu H., Liu Y., Sun F. (2014). Traffic sign recognition using group sparse coding. Inf. Sci..

[19] Yang J., Wang Z., Lin Z., Cohen S., Huang T. (2012). Coupled dictionary training for image super-resolution. IEEE Trans. Image Process..

[20] Liu H., Yu Y., Sun F., Gu J. (2017). Visual-tactile fusion for object recognition. IEEE Trans. Autom. Sci. Eng..

[21] Liu H., Liu Y., Sun F. (2015). Robust exemplar extraction using structured sparse coding. IEEE Trans. Neural Networ..

[22] Qiao T., Li W., Wu B., Wang J. (2013). A chaotic iterative algorithm based on linearized Bregman iteration for image deblurring. Inf. Sci..

[23] Liu H., Guo D., Sun F. (2016). Object recognition using tactile measurements: Kernel sparse coding methods. IEEE Trans. Instrum. Meas..

[24] Zhu Z., Chai Y., Yin H., Li Y., Liu Z. (2016). A novel dictionary learning approach for multi-modality medical image fusion. Neurocomputing.

[25] Yang B., Li S. (2010). Multifocus image fusion and restoration with sparse representation. IEEE Trans. Instrum. Meas..

[26] Li S., Yin H., Fang L. (2012). Group-sparse representation with dictionary learning for medical image denoising and fusion. IEEE Trans. Biomed. Eng..

[27] Yang B., Li S. (2012). Pixel-level image fusion with simultaneous orthogonal matching pursuit. Inf. Fusion.

[28] Wang K., Qi G., Zhu Z., Chai Y. (2017). A novel geometric dictionary construction approach for sparse representation based image fusion. Entropy.

[29] Nejati M., Samavi S., Shirani S. (2015). Multi-focus image fusion using dictionary-based sparse representation. Inf. Fusion.

[30] Yin H., Li Y., Chai Y., Liu Z., Zhu Z. (2016). A novel sparse-representation-based multi-focus image fusion approach. Neurocomputing.

[31] Kim M., Han D.K., Ko H. (2016). Joint patch clustering-based dictionary learning for multimodal image fusion. Inf. Fusion.

[32] Meskine F., Mezouar M.C.E., Taleb N. (2010). A Rigid image registration based on the nonsubsampled contourlet transform and genetic algorithms. Sensors.

[33] Li H., Chai Y., Li Z. (2013). Multi-focus image fusion based on nonsubsampled contourlet transform and focused regions detection. Optik.

[34] Li X., Li H., Yu Z., Kong Y. (2015). Multifocus image fusion scheme based on the multiscale curvature in nonsubsampled contourlet transform domain. Opt. Eng..

[35] Rubinstein R., Zibulevsky M., Elad M. (2010). Double sparsity: Learning sparse dictionaries for sparse signal approximation. IEEE Trans. Signal Process..

[36] Dong W., Zhang L., Lukac R., Shi G. (2013). Sparse representation based image interpolation with nonlocal autoregressive modeling. IEEE Trans. Signal Process..

[37] Peleg T., Elad M. (2014). A statistical prediction model based on sparse representations for single image super-resolution. IEEE Trans. Signal Process..

[38] Liu Z., Blasch E., Xue Z., Zhao J., Laganiere R., Wu W. (2012). Objective assessment of multiresolution image fusion algorithms for context enhancement in night vision: A comparative study. IEEE Trans. Pattern Anal. Mach. Intell..

[39] Nava R., Cristóbal G., Escalanter-amírez B. (2007). Mutual Information Improves Image Fusion Quality Assessments. Electron. Imaging Signal Process..

[40] Yang C., Zhang J.Q., Wang X.R., Liu X. (2008). A novel similarity based quality metric for image fusion. Inf. Fusion.

[41] Petrović V. (2007). Subjective tests for image fusion evaluation and objective metric validation. Inf. Fusion.

[42] Zhao J., Laganiere R., Liu Z. (2007). Performance assessment of combinative pixel-level image fusion based on an absolute feature measurement. Int. J. Innov. Comput. I..

[43] Qu G., Zhang D., Yan P. (2002). Information measure for performance of image fusion. Electron. Lett..

[44] Chen Y., Blum R.S. (2009). A new automated quality assessment algorithm for image fusion. Image Vis. Comput..

[45] Sheikh H.R., Bovik A.C. (2006). Image information and visual quality. IEEE Trans. Image Process..

[46] Kim M., Han D.K., Ko H. Multimodal image fusion via sparse representation with local patch dictionaries. Proceedings of the IEEE International Conference on Image Processing.

[47] Zhu Z.Q., Yin H., Chai Y., Li Y., Qi G. (2017). A novel multi-modality image fusion method based on image decomposition and sparse representation. Inf. Sci..

[48] Shao Z., Jun L., Qimin C. (2012). Fusion of infrared and visible images based on focus measure operators in the curvelet domain. Appl. Opt..

[49] Shah P., Merchant S.N., Desai U.B. (2010). Fusion of surveillance images in infrared and visible band using curvelet, wavelet and wavelet packet transform. Int. J. Wavelets Multiresolut. Inf. Process..

